# Roles of Histone Acetylation and Deacetylation in Root Development

**DOI:** 10.3390/plants13192760

**Published:** 2024-10-01

**Authors:** Christos Tersenidis, Stylianos Poulios, George Komis, Emmanuel Panteris, Konstantinos Vlachonasios

**Affiliations:** 1 Department of Botany, School of Biology, Aristotle University of Thessaloniki, 54124 Thessaloniki, Greece; ctersen@bio.auth.gr (C.T.); spoulios@bio.auth.gr (S.P.); gkomis@bio.auth.gr (G.K.); epanter@bio.auth.gr (E.P.); 2 Natural Products Research Centre of Excellence (NatPro-AUTh), Center of Interdisciplinary Research and Innovation, Aristotle University of Thessaloniki (CIRI-AUTh), 57001 Thessaloniki, Greece

**Keywords:** epigenetic modifications, histone acetylation, histone deacetylation, root development, gene expression, quiescent center maintenance, cell division, mitosis, cell elongation, epidermis cell fate

## Abstract

Roots are usually underground plant organs, responsible for anchoring to the soil, absorbing water and nutrients, and interacting with the rhizosphere. During root development, roots respond to a variety of environmental signals, contributing to plant survival. Histone post-translational modifications play essential roles in gene expression regulation, contributing to plant responses to environmental cues. Histone acetylation is one of the most studied post-translational modifications, regulating numerous genes involved in various biological processes, including development and stress responses. Although the effect of histone acetylation on plant responses to biotic and abiotic stimuli has been extensively reviewed, no recent reviews exist focusing on root development regulation by histone acetylation. Therefore, this review brings together all the knowledge about the impact of histone acetylation on root development in several plant species, mainly focusing on *Arabidopsis thaliana*. Here, we summarize the role of histone acetylation and deacetylation in numerous aspects of root development, such as stem cell niche maintenance, cell division, expansion and differentiation, and developmental zone determination. We also emphasize the gaps in current knowledge and propose new perspectives for research toward deeply understanding the role of histone acetylation in root development.

## 1. Introduction

Roots are usually underground plant organs that, in addition to providing structural support to the aerial parts of the plant, are responsible for water and nutrient uptake as well as plant–microbe interactions [1]. Root development can be influenced by various environmental signals, and proper root growth and function are crucial for overall plant survival [2,3]. Root cells can respond to external signals by altering gene expression in response to environmental cues [2,3,4]. DNA in the nucleus is wrapped around histone octamers to form nucleosomes, the core chromatin particles [5,6]. Chromatin structure is versatile and is determined by multiple mechanisms, including—amongst others—DNA methylation, histone modifications, ATP-dependent chromatin remodeling, and incorporation of histone variants [7,8,9,10,11]. In particular, N-terminal tails of histone proteins can be subjected to methylation, acetylation, phosphorylation, ubiquitination, and sumoylation, which can have synergistic or antagonistic effects [7]. Collectively, these post-translational histone modifications are referred to as the “histone code” and are pivotal for regulating gene expression [12].

One of the most studied histone post-translational modifications is histone acetylation, which is a fundamental and conserved process that controls chromatin structure and regulates gene expression, significantly influencing most aspects of plant development [13,14]. Increasing evidence supports that histone acetylation and deacetylation are involved in the regulation of gene expression, not only during plant development but also during plant responses to environmental stimuli [13,14,15,16,17,18]. The latter has been extensively reviewed both in the past and in the present [19,20,21,22,23,24]; however, there are no recent reviews that clearly focus on the roles of histone acetylation during root development. Hence, the purpose of this review is to summarize all the available knowledge around the impact of histone acetylation on root development of several plant species, mainly focusing on the primary root of *Arabidopsis thaliana*. This information will contribute to our understanding of the molecular mechanisms of gene expression regulation by histone acetylation during root growth and differentiation.

## 2. The *Arabidopsis thaliana* Primary Root

Studies on root development have mainly focused on the model organism *A. thaliana*, because of its advantages in comparison to other plant species: its relatively simple root cell patterning, combined with amenability for experimental manipulations, have established it as a powerful tool for studying developmental processes [25]. Over the past 35 years, “classic” anatomical studies, genetic experiments, and modern molecular biology and genomic techniques have revealed the cellular organization of the *A. thaliana* primary root and the control of root development at the molecular level [26]. In order to discuss and highlight the impact of histone acetylation on root development, a summary of some key elements regarding the organization of *A. thaliana* primary root must be provided.

Primary root meristem organization in *A. thaliana* is established during embryogenesis as the pattern of cell divisions in the embryo gives rise to the various initial cells within the root promeristem [27]. Post-embryonic root organization is apparent in the mature embryo and is maintained in the growing primary root after germination. The quiescent center (QC) consists of four hypophysis-derived cells, located between the root cap columella and the stele, and acts as a “cytogenerative centre” [28,29]. The QC cells are surrounded by the initial cells and they comprise the stem cell niche (SCN), which give rise to the root cell files: the stele, the cortex and endodermis, the epidermis and lateral root cap, and the columella [28,30].

Maintenance and homeostasis of the SCN in *A. thaliana* root are essential for the growth and development of all root cell types. WUSCHEL-RELATED HOMEOBOX 5 (WOX5) and PLETHORAs (PLTs) are critical transcription factors that are expressed in the SCN, where they maintain the QC and regulate distal columella stem cell fate [31]. Two parallel pathways, the PLT [32,33] and the SHORT-ROOT (SHR)/SCARECROW (SCR)/RETINOBLASTOMA-RELATED (RBR) pathway [34,35,36,37], are responsible for the specification of the SCN in the root.

From a geometrical point of view, *A. thaliana* root can be viewed as a set of concentric cylinders. From outward to inward, the epidermis, cortex, endodermis, and pericycle layers surround the vascular tissue in the middle of the root [38]. The number of cells in each layer and their location in the primary root are invariable; the cortex and the endodermis comprise eight cell files, whereas the cell number in the epidermis and pericycle is more variable [28]. Root epidermis, also referred to as the rhizodermis, is the outermost layer, which is in contact with the environment. Specialized cells in the epidermis form root hairs that anchor roots to the soil, absorb water and nutrients, and are responsible for plant-microbe interactions, while other cells have a non-hair phenotype [39,40]. Next centripetally, the ground tissue consists of the cortex and endodermis. The cortex is involved in determining epidermal cell fate, transporting water and nutrients into the vasculature, and storing substances such as starch, resins, and essential oils [41,42]. The endodermis acts as an apoplastic barrier during the uptake and radial transport of water and solutes by developing the Casparian strip; it also mechanically supports the stele and protects it against pathogens and parasites [26,42]. The stele is located in the middle of the root and consists of the pericycle and the vascular tissue [43]. The pericycle is responsible for the production of lateral roots, vascular cambium, and periderm, while the vasculature provides mechanical support, nutrient/water transport, and produces signaling molecules [44,45].

The root apex of *A. thaliana* exhibits a basipetal arrangement of four distinct developmental zones of increasing maturity: the meristematic zone, the transition zone, the elongation zone, and the differentiation zone. These zones are well-defined, based on their characteristic cellular activities. The meristematic zone is characterized by small, undifferentiated, mitotically active cells. Immediately shootward after the meristematic zone, the transition zone consists of cells preparing for fast elongation through physiological changes, such as development of a central vacuole, polarization of the cytoskeleton, and cell wall remodeling. In the transition zone, cells also acquire high sensitivity to diverse environmental factors, which can affect both the signal-mediated tropisms and root morphogenesis [46]. Root development is highly responsive to altering environmental conditions and the transition zone acts as a kind of “dynamic reservoir”, including developmentally plastic cells, which rapidly adjust their growth speed and direction in response to the environmental conditions [26,46]. Next shootward, cells in the elongation zone undergo fast anisotropic diffuse growth before entering the differentiation zone, where they follow their ultimate differentiation pathway [46,47]. Overall, plants integrate hormone signaling, nutrient availability, and several other external cues to regulate several aspects of root development, such as cell proliferation, elongation, and differentiation, determining their ability to adapt to the environmental conditions [3,48,49].

## 3. Histone Acetylation in *Arabidopsis thaliana*: A Dynamic and Reversible Process

Eukaryotic genomic DNA is wrapped around histones, forming the nucleosomes, the core particles of chromatin. Nucleosome conformations produce higher-order chromatin structures, packing DNA into the cell nucleus [50]. Chromatin conformation and nucleosome organization critically affect gene expression modulation, as it depends on the DNA’s accessibility to transcription factors, DNA-binding activators, and modifying enzymes [13]. Chromatin remodeling leads to the reconfiguration of protein–DNA interactions and is linked to changes in genomic activity, such as gene expression. Chromatin remodeling can be achieved by a diverse array of mechanisms; however, in this review, focus is placed on post-translational modifications of chromatin components, in particular histone acetylation [13,51].

Histone N-terminal tails protrude from the nucleosomes and can be post-translationally acetylated, methylated, phosphorylated, ubiquitinated, glycosylated, ADP ribosylated, carbonylated, sumoylated, and biotinylated [52,53,54]. Distinct histone amino-terminal modifications can generate synergistic or antagonistic effects, dictating the level of transcriptional activity. Collectively, all these modifications are referred to as the “histone code”, affecting a plethora of biological processes in response to internal and external cues [3,52,53,54,55]. Histone acetylation, in particular, plays a role in hormone signaling, seed development, dormancy and germination, root and leaf development, regulation of flowering time, floral meristem function, flower fertility, photomorphogenesis, and biotic and abiotic stress responses [15,16,17,20,21,23,55,56,57,58,59,60,61,62,63,64,65,66,67,68,69,70,71]. It therefore becomes evident that histone modifications contribute to the higher developmental plasticity of plants and allow them to respond rapidly to environmental signals by chromatin-mediated control of gene expression [3,22].

Histone acetyltransferases (HATs) and deacetylases (HDAs) modify histone N-tails. HATs transfer acetyl groups to lysine residues, while HDAs catalyze the opposite reaction of acetyl group removal. HATs and HDAs are usually active in the nucleus but there are some members that are found in the cytoplasm as well [72]. Acetylation of lysine residues neutralizes the positive charge of histones, increasing chromatin accessibility and allowing the binding of cis- or trans-acting factors and the regulation of transcriptional activity [14,50]. Highly acetylated histones are associated with transcriptionally active genes, whereas deacetylated histones are linked to transcriptional repression [73,74]. HATs are constituents of large multiprotein complexes, such as the SAGA complex, where they interact with activators, transcription factors, and chromatin modulators and are recruited to genomic target sequences, promoting either the activation or suppression of gene expression [58,75,76]. Most of the HDA proteins, as well, interact with some other co-factors to assemble multiprotein complexes, such as the Sin3-HDA complex [77,78].

The homeostasis of histone acetylation in vivo is dynamically maintained by HATs and HDAs [13]. *A. thaliana* HATs are categorized into four families: the GCN5-RELATED N-TERMINAL ACETYLTRANSFERASES (GNATs); the MYST (MOZ, Ybf2/Sas3, Sas2, and Tip60)-related HATs; p300/CREB-binding protein (CBP) HATs; and TRANSCRIPTION INITIATION FACTOR TAF_II_250 (for TATA-d factor). On the other hand, HDAs include three families: the REDUCED POTASSIUM DEPENDENCY 3/HISTONE DEACETYLASE 1 (RPD3/HDA1) superfamily; the SILENT INFORMATION REGULATOR 2 (SIR2) family; and the plant-specific HISTONE DEACETYLASE 2 (HD2) family [79]. Even though *A. thaliana* has numerous HATs and HDAs, not all HAT and HDA families have been studied to the same extent and not all members across each family have been investigated equally regarding their contribution to root development [58,80,81,82].

An example of a well-studied histone acetyltransferase is the GENERAL CONTROL NON-DEREPRESSIBLE PROTEIN 5 (GCN5), which belongs to the GNAT family [58]. It acts as a transcriptional adaptor in the HAT module (HATm) of two distinct transcriptional adaptor complexes in *A. thaliana*, the well-known SAGA (Spt-Ada-Gcn5-acetyltransferase) and the recently discovered plant-specific PAGA complex [58,76,83]. The GCN5-containing complexes can acetylate histones in nucleosomes [84] and are conserved in many eukaryotes [85]. The ALTERATION/DEFICIENCY IN ACTIVATION 2b (ADA2b) protein is another HATm subunit, which interacts with GCN5 and acts as a transcriptional co-activator. In multicellular eukaryotes, GCN5-containing complexes appear to play essential developmental roles [86]. In *A. thaliana*, *gcn5* and *ada2b* mutants exhibit pleiotropic defects in every developmental procedure, such as leaf development, apical dominance, inflorescence development, floral meristem function, and flower fertility [56,57,58,59,62,63,67,87], including defects in root growth [17,59,61,88].

## 4. Histone Acetylation Is Required for QC Specification, Stem Cell Niche Maintenance, and Cell Proliferation in the Meristematic Zone

**Figure 1:**
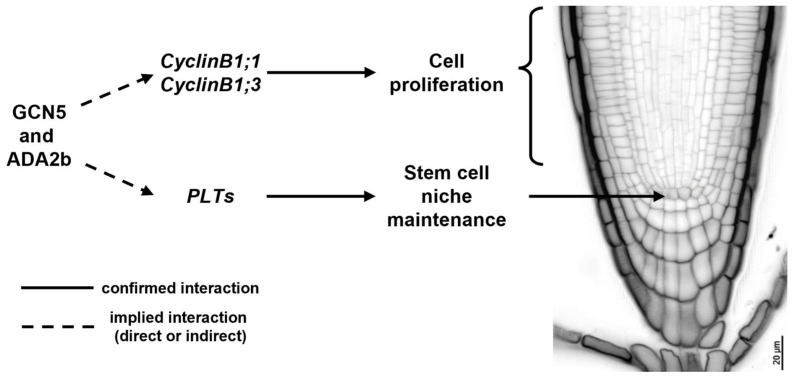
The effect of GCN5 and ADA2b on cell proliferation and stem cell niche maintenance in *Arabidopsis thaliana* wild-type roots.

Histone acetylation is necessary for SCN specification as wild-type *A. thaliana* roots treated with trichostatin A (TSA), a specific HDA inhibitor, form extra layers of ground tissue (premature middle cortex formation), exhibit abnormal cell divisions in the QC and surrounding stem cells, and result in decreased root growth [89]. More specifically, histone acetylation complexes could shape a developmentally instructive gradient, responsible for the specification of the SCN in the root, such as the PLT gradient [32,33]. Indeed, GCN5 is essential for root SCN maintenance and acts in the PLT pathway alongside ADA2b [68]. GCN5 and ADA2b act independently of the SHR/SCR/RBR pathway, and they positively regulate the expression of *PLTs* and mediate the proliferation of QC neighboring cells, as indicated by the *gcn5* and *ada2b* mutants, which result in perturbed QC specification and a smaller meristem [59,68]. Besides QC specification, GCN5 and ADA2b modulate stem cell proliferation of transit-amplifying cells and, therefore, meristem size [68]. GCN5 and ADA2b regulate the expression of several genes involved in cell cycle progression, such as *CYCLINB1* (Figure 1) [68,88]. Moreover, ADA2b, also known as PROPORZ1 (PRZ1), acts as a developmental switch from cell proliferation to differentiation, in response to fluctuations in auxin and cytokinin [88]. In particular, ADA2b’s effect on cell proliferation can be mediated by positively regulating the expression of several cell cycle regulators, such as *B*- and *D-type CYCLINS* and *KIP-RELATED PROTEIN* (*KRP*) members [88,90]. Although the role of histone acetylation in cell cycle progression and QC specification becomes evident, it still remains unclear whether the aforementioned genes are directly regulated by histone acetylation patterns in their regulatory regions. The only available data can be traced back to research related to callus formation, where GCN5 catalyzes histone acetylation at the *WOX5*, *WOX14*, *SCR*, *PLT1*, and *PLT2* regulatory regions, epigenetically regulating their transcriptional activation [91]. However, no data are available regarding the in planta regulation of key developmental players for QC specification and maintenance and cell cycle progression.

## 5. Histone Acetylation Has a Fine-Tuning Role in Root Elongation

Epigenetic regulation may control root development through auxin signaling. The widely used HDA inhibitors, sodium butyrate and TSA, inhibit primary root elongation and lateral root emergence in wild-type *A. thaliana* plants. In response to the treatment, the auxin efflux transporter protein PIN-FORMED 1 (*PIN1*) is degraded by the 26S proteasome in the root tip, and auxin distribution is altered. *PIN1* gene expression is not altered in the presence of the inhibitors, whereas transcription of *IAA* genes increases [92]. However, the expression of *PIN1* and several other PIN genes are altered in *gcn5* and *ada2b* mutants, indicating that GCN5 and ADA2b act as positive regulators of auxin distribution in early *A. thaliana* root growth [61].

In addition, the plant-specific histone deacetylases HISTONE DEACETYLASE 2A HDT1 (HD2A or HDT1) and 2B (HD2B or HDT2) regulate *GIBBERELLIN 2-OXIDASE2* (*GA2OX2*) expression, controlling the number of *A. thaliana* root meristem cells. By repressing the expression of *GA2OX2*, *HDT1* and *HDT2* act as fine-tuners of gibberellin metabolism and regulate the transition from cell division to cell expansion. Two plant-specific histone deacetylases are proposed to modulate root growth in response to environmental factors [93].

Histone acetylation can modulate root elongation via a transcriptional repressor, LYSINE-SPECIFIC HISTONE DEMETHYLASE 1 (LDL1/SWP1), which negatively regulates the expression of the root-specific *LATERAL ROOT PRIMORDIUM 1* (*LRP1*) gene by H3 and H4 deacetylation. It seems like LDL1 functions in a repressor complex that includes HDA(s) and a putative DNA-binding protein to target the LRP1 promoter. The extent of this regulation in root length is modest, suggesting that it represents a mechanism for fine-tuning root elongation [94].

HISTONE DEACETYLASE 19 (HDA19) can also control root cell elongation and modulate a subset of phosphate starvation responses in *A. thaliana*. Through an interplay of Pi availability and intrinsic factors, HDA19 controls the cell length of epidermal cells, probably by altering the positional bias that dictates epidermal patterning [95].

## 6. Histone Acetylation Modulates Cell Differentiation during Root Development

### 6.1. Histone Acetylation Regulates Columella Cell Differentiation in the Root Cap

In *A. thaliana* root meristem, the columella stem cells (CSCs) are located distally to the QC and undergo asymmetric divisions, generating descendants that immediately differentiate and accumulate gravity-sensing amyloplasts [28]. WOX5 moves from the QC into the CSCs and maintains the undifferentiated state of the CSCs [96]. In the CSCs, WOX5 recruits TOPLESS/TOPLESS-RELATED (TPL/TPR) co-repressors and HDA19 and directly represses the expression of *CYCLING DOF FACTOR 4* (*CDF4*) by inducing histone deacetylation in the *CDF4 cis*-regulatory region. As a result, a gradient of *CDF4* transcription is created, opposite to the WOX5 gradient, enabling the columella stem cell daughter cells to exit the stem cell state and enter a differentiation state while simultaneously preventing the differentiation of the QC cells and CSCs [97]. This is one characteristic example of histone acetylation-mediated repression of differentiation programs in specific cells during development through regulating the transcriptional profile of a specific gene.

### 6.2. Histone Acetylation Regulates Cell Patterning in the Root Epidermis

The mature epidermis of *A. thaliana* comprises two cell types: trichoblasts that give rise to root hairs, and atrichoblasts. These cells are arranged in sixteen or more discrete and alternating files. Each hair cell file overlies the anticlinal/radial wall of the underlying cortex cells (hair position or H position) and is separated from the next hair cell file by one or more non-hair files, which are located over the outer periclinal/tangential wall of the underlying cortex cells (non-hair position or N position). Since the cortex comprises eight cell files, the trichoblasts are always arranged into eight files as well and the root hairs emerge at the differentiation zone as tip-growing projections from the rootward end of the trichoblasts [30].

The position-dependent cellular pattern of epidermal cells is determined by interactions of six major patterning genes: *GLABRA 2* (*GL2*)*, GLABRA 3* (*GL3*)*, CAPRICE* (*CPC*)*, ENHANCER OF TRY AND CPC 1* (*ETC1*)*, TRANSPARENT TESTA GLABRA 1* (*TTG1*), and *WEREWOLF* (*WER*). Trichoblast and atrichoblast cell fate results from intra- and intercellular position-dependent signaling and complex feedback loops that ultimately define *GL2*-expressing and non-expressing cells. The expression of the *GL2* transcription factor is required to specify the atrichoblast cell fate. The position-dependent expression of the *CPC*, *GL2*, and *WER* genes is essential for their appropriate interactions [98].

TSA treatment in wild-type *A. thaliana* plants alters the cell patterning of the root epidermis, inducing hair cell development at non-hair cell positions. The effect of TSA is rapid, reversible, concentration-dependent, and position-independent. These findings suggest that histone acetylation may mediate a positional cue to direct expression of the patterning genes in root epidermal cells. Therefore, it is established that cell patterning of the root epidermis can be affected by histone H3 and H4 hyperacetylation and ectopic expression of *CPC*, *GL2*, and *WER* [99].

In addition, mutants of the *HISTONE DEACETYLASE 18* (*HDA18*) exhibit altered H and N epidermal cell patterning, similar to that observed in TSA treatment experiments, highlighting that HDA18 is required for the establishment of root epidermal cell patterning by regulating histone acetylation. Overexpression of *HDA18* results in the same phenotype, which indicates that epidermal cell identity depends on a balanced histone acetylation profile. However, contrary to TSA treatment experiments, no patterning genes are affected in *hda18* mutants. Instead, several kinase loci are regulated by HDA18 at a transcriptional level through histone acetylation [99,100].

Therefore, it becomes evident that cell patterning in the *A. thaliana* root epidermis depends on transmitting positional information through histone acetylation. This notion is further supported by the loss-of-function mutants of *HISTONE DEACETYLASE 6* (*HDA6*). Similarly to *hda18* mutants, *hda6* mutants exhibit ectopic root hairs at the N positions. HDA6 is found to be directly bound to the promoter regions of *ETC1* and *GL2*, regulating both the transcript levels and expression patterns of these genes in the root tip by altering the histone acetylation status of their promoter regions. Contrary to *HDA18*, overexpression of *HDA6* does not interfere with the cell patterning phenotype. In this way, HDA6 is characterized as a regulator of the transcription of two core factors involved in determining epidermal cell fate [101].

The phenotypic analysis of all the available HDA and HAT single mutants highlighted one additional histone deacetylase (*HDA19*) and two histone acetyltransferase (*GCN5* and *HISTONE ACETYLTRANSFERASE OF THE TAFII250 FAMILY 2, HAF2*) mutants with altered cell patterns in root epidermis, exhibiting ectopic root hair formation at the N positions. Based on these results, it is proposed that histone acetylation homeostasis is important for the robustness of the regulatory network responsible for cell patterning in *A. thaliana* root epidermis [102].

HDA19 and SCR are reported to regulate cortex cell fate, concomitantly affecting root epidermis cell patterning. HDA19 regulates the expression of *SCR* and several SCR target genes by binding to the DNA sequence upstream of them. *HDA19* overexpression lines did not cause any dramatic changes to the epidermis or cortex patterning [103].

It is also known that during trichome development, GCN5 regulates the expression of the core genes *CPC*, *GL1*, *GL2*, and *GL3* through histone acetylation. GCN5 can bind directly to the promoter region of *GL2* to regulate its expression, as does *HDA6* [67,101]; however, it remains to be seen whether GCN5 acts the same way in the root and affects cell patterning of the root epidermis.

### 6.3. Histone Acetylation Regulates Lateral Root Development

Lateral roots arise from the pericycle by periclinal and anticlinal divisions and are formed at a distance from the primary root meristem. Lateral root cell organization is similar to that of the primary root but it is characterized by more variability in the number of cell files in each cell layer [28]. Lateral root formation determines root system architecture, and the degree of root branching affects both the efficiency of water and nutrient uptake and anchorage to the soil [104]. Even though the regulation of lateral root development is of high agronomic importance, the effect of histone acetylation on it remains poorly understood.

As mentioned above, HDA inhibitors repress primary root elongation and lateral root emergence in wild-type *A. thaliana* [92]. This indicates that HDA activity positively regulates lateral root emergence. Furthermore, TSA treatment alters the expression of S-*PHASE KINASE-ASSOCIATED PROTEIN 2B* (*SKP2B*), which regulates the stability of cyclin-dependent kinase inhibitor KRP1. *skp2b* mutant exhibits increased root elongation and lateral root emergence, supporting that SKP2B negatively regulates cell cycle and lateral root formation. The promoter region of SKP2B is regulated by H3 acetylation in an auxin- and IAA14-dependent manner, supporting the view that epigenetics represents an important regulatory mechanism during lateral root formation [105].

Auxin signaling and variations in *KRP* expression have been suggested to be important for the control of lateral root formation [106,107]. Auxin treatment triggers formation of callus-like tissue on *prz1-1* roots instead of promoting lateral root formation. KRPs are core cell cycle regulators and their disproportionate expression reduction in *prz1-1* is proposed to affect initiation and further differentiation of lateral roots, suggesting that ADA2b can also control lateral root formation [88]; however, this proposition has not been verified yet.

As *swp1* mutants have increased lateral root density and length, SWP1 is a negative regulator of lateral root initiation and elongation through direct/indirect transcriptional repression of *AUXIN RESPONSE FACTORS* (*ARFs*) and *GATA23*, which are lateral root-promoting factors [108]. Since SWP1 negatively regulates the expression of *LRP1* by H3/H4 deacetylation and modulates root elongation [94], it also suggested that SWP1 could regulate lateral root growth by repressing *LRP1*, as in the primary root. The possibility for LRP1 or an unknown factor to act downstream of SWP1 to negatively regulate auxin-signaling and ARF7/ARF19-mediated lateral root formation is also mentioned [108]. More recent findings support that *LRP1* expression is affected by histone deacetylation and auxin and that LRP1 negatively regulates lateral root primordium development by modulating auxin homeostasis. LRP1 acts downstream of auxin-responsive Aux/IAA-ARF modules during lateral root development and an SWP1/HDA-containing corepressor complex may be involved in HDA-mediated repression of *LRP1* required for lateral development [109]. It is established that histone deacetylation has an impact on auxin-dependent expression of *LRP1* during lateral development but it is still unknown which HDA interacts with SWP1 during lateral development. HDA6 could be a good candidate for this interaction since it has been shown to interact with SWP1 to regulate circadian clock genes [110].

Plant-specific histone deacetylases are also involved in lateral root development. More specifically, overexpression of *HISTONE DEACETYLASE 2D* (*HD2D*) leads to the formation of more and longer lateral roots [111]. This was the first indication that HD2D is a positive regulator of lateral root emergence and development, verifying the TSA observations. Recently, it was suggested that the overexpression of *HD2D* leads to ROS accumulation and ROS-mediated lateral root development, giving hints about the mechanism that might be affected [112]. The CKII phosphorylation of HD2D was later shown to contribute to lateral root development and abscisic acid (ABA) sensing in *A. thaliana* [113]. HD2D was also recently shown to regulate the gene transcription levels required to maintain the root tip microenvironment. In addition, it was confirmed that HD2D is involved in regulating lateral root development, while the possibility of auxin-mediated regulation of lateral root development was further explained. By promoting lateral root development, HD2D could be involved in coping with abiotic stress, effectively enhancing the resistance of *A. thaliana* to it [112].

## 7. Histone Acetylation Modulates Root Zonation

*PLTs* are expressed in the meristematic zone and form a gradient with a maximum concentration near the root tip. This gradient acts as a dose-dependent master regulator of *A. thaliana* root development and is a prerequisite for the zonation of roots. High levels of *PLT* expression promote stem cell maintenance, intermediate levels enhance mitotic activity, and low *PLT* expression leads to cell elongation and differentiation [32,114,115].

As stated above, GCN5 and ADA2b act in the *PLT* pathway, suggesting that the expression of *PLTs* is positively controlled by histone acetylation [68]. Therefore, histone acetylation-mediated control of *PLT* expression is essential for root zonation. However, it remains uncertain whether the expression of *PLT* genes is directly regulated by GCN5 histone acetylation. Most evidence indicates an indirect way of regulating the expression of *PLTs* by GCN5. ChIP-chip analysis in seedlings with anti-GCN5 antibodies revealed that GCN5 does not associate with *PLT* genes [116]. In addition, *PLT*2 promoter H3K9ac/K14ac levels are sustained in *ada2b* mutants, suggesting a possibility of GCN5-independent histone acetylation [90]. On the other hand, the only finding that associates GCN5 with histone acetylation at the *PLT*1 and *PLT*2 regulatory regions derives from a study regarding callus formation and pluripotency acquisition, while no in planta verification is yet available [92].

Since *PLTs* form a gradient of expression in the root apex, and GCN5 and ADA2b are ubiquitously expressed [32,68], *PLT* expression can be indirectly affected by factors downstream of GCN5 that also form a gradient. In addition, since auxin is involved in *PLT* expression and is accumulated in the SCN [32,114,115], GCN5 may control the expression of some auxin-related factors, which in turn indirectly affect the *PLT* gradient in the root tip. This hypothesis can be supported by the fact that GCN5 acts as a positive regulator of auxin distribution in early root growth by modulating histone H3 acetylation and the expression of PIN auxin efflux transport genes [61]. GCN5-mediated regulation of auxin distribution could indirectly affect the expression of *PLT* genes and the *PLT* gradient in the RAM. Also, in the presence of auxin, bZIP11-related basic leucine zipper (bZIP) transcription factors bind to the promoter regions of auxin-responsive genes and recruit histone acetylation machinery by interacting with ADA2b. In this way, bZIPs can directly bind to the *GH3.3* promoter and recruit GCN5 via ADA2b to activate the expression of *GH3.3*. This may boost the expression of auxin-responsive genes, and such a link between the GCN5-containing complex and auxin-related factors may explain the consequent formation of the *PLT* gradient in the root tip [117]. Current research has not yet provided a solid explanation about the direct or indirect regulation of *PLTs* expression by GCN5, and the mentioned hypothesis seems weak without experimental verification. Future research could add mechanistic support to the hypothesis, which needs to be further tested in order to be verified or declined.

## 8. The Effect of Histone Acetylation and Deacetylation on Root Development in *Oryza sativa*, *Zea mays,* and *Populus trichocarpa*

Apart from *A. thaliana* primary root, the purpose of this review is to summarize all the available knowledge around the impact of histone acetylation on root development of several plant species. This section will discuss histone acetylation in other model plant species, such as the monocots *Oryza sativa* and *Zea mays*, and *Populus trichocarpa*, a woody plant. As in *A. thaliana*, the HAT genes are grouped into four families (GNAT, MYST, CBP, and TAF_II_250) and the HDAs into three (RPD3/HDA1, SIR2, and HD2); however, the number of members in each family varies amongst the species (Table 1).

The rice (*O. sativa*) genome encodes for 8 HAT and 18 HDA genes [118,119]. Expression analysis showed that all genes are expressed in all tissues, though with different abundances in each [119]. The *AtGCN5* homolog of rice is *OsHAG702*, which has been found to be heat- and ABA-inducible as well as cold-suppressible [119]. Some HDA genes are expressed in an organ-specific pattern, like the *OsHDA705* and *OsHDT702/HISTONE DEACETYLASE 1* (*OsHDAC1*) that are specifically expressed in seedling roots [118]. Several rice *HDA* genes have been shown to be differentially expressed in response to environmental stressors, such as drought, salt, and cold. These genes are also responsive to phytohormones, such as jasmonic acid (JA), salicylic acid (SA), and ABA, which are key players in mediating both abiotic and immune responses [20,21,23]. While histone acetylation in rice has been studied during plant responses, few data are available regarding root development.

In *O. sativa* roots, contrary to *A. thaliana*, hypo-acetylation leads to the opposite phenotype, which indicates that HDAs might have divergent functions depending on the plant species. In particular, overexpression of the class I-type *OsHDAC1* correlates with an increase in HDA activity, reduction in acetylation of H4, and higher seminal root growth rate [120]. OsHDAC1 seems to epigenetically regulate the *OsNAC6* gene, which in turn regulates root growth. OsHDAC1-mediated deacetylation of K9, K14, and K18 on histone H3 and K5, K12, and K16 on histone H4 results in an altered histone acetylation profile in the *OsNAC6* promoter region and regulates the transcription rate of the gene. *OsNAC6* knockout seedlings exhibit similar root phenotypes to that of the *OsHDAC1* overexpression seedlings, further supporting that *OsNAC6* is the critical component of the OsHDAC1 regulon [121]. OsHDAC1 is also a positive regulator of lateral root formation since *OsHDAC1 RNAi* plants produced fewer lateral roots, whereas *OsHDAC1* overexpression lines exhibit increased lateral root primordia. It is now known that OsHDAC1 can directly interact with and deacetylate GSK3/SHAGGY-LIKE KINASE 2 (OsGSK2), inhibiting its activity to phosphorylate its substrates. The deacetylated OsGSK2 has an attenuated interaction with BRASSINAZOLE-RESISTANT 1 (OsBZR1), a positive regulator of lateral root primordium formation. As a result, OsBZR1 is accumulated, and higher lateral root formation is observed by regulating Auxin/IAA signaling genes. Therefore, lateral root formation in rice is controlled by the HDAC1/GSK2/BZR1 module [122].

WUSCHEL (WUS)-RELATED HOMEOBOX11 (OsWOX11) is necessary and sufficient to promote rice crown root emergence and elongation. *OsADA2* and *OsGCN5* genes are highly expressed in root meristem and are essential for cell division and growth. OsWOX11 recruits the ADA2-GCN5 histone acetyltransferase module to activate the transcription of downstream root-specific target genes in the crown root meristem. These target genes are involved in energy metabolism, cell wall biosynthesis, and hormone response, which are essential for root development [123]. In addition, OsWOX11 can maintain reactive oxygen species (ROS) homeostasis by upregulating peroxidase genes in the crown root meristem. It appears that the redox state affects HDA activity, which results in high levels of protein lysine acetylation in crown root cells [124].

Our understanding of histone acetylation in maize (*Z. mays*) is significantly inferior compared to other plant species. Maize has 18 HDA genes and expression analysis has shown that most of them have a widespread expression pattern but with differences in their abundance; for example, the genes *ZmHD2B* (GRMZM2G100146) and *ZmHD2C* (GRMZM2G159032) are highly expressed in the primary root, whereas the gene GRMZM5G807054 is significantly downregulated in the same organ [125]. As is the case for rice, abiotic stresses and hormones affect the expression of HDA genes; SA and Methyl-Jasmonate (MeJA) downregulated most of the genes in a time course experiment but some genes were induced. Most of the genes also were induced under drought, UV, heat, cold, and salt stresses. Alterations in acetylation levels of H3K9 and H4K5 upon cold and heat stress treatment suggest that maize HDAs might be involved in abiotic stress responses by regulating histone acetylation levels [18, 125]. Even less is known about *Z. mays* HATs. More than 30 years ago, two forms of nuclear HATs, *ZmHAT-A1* and *ZmHAT-A2*, were identified, each one specialized for H3-H4 and H3, respectively [126], and involved in seed and embryo germination [127]. In maize, one *AtGCN5* homolog has been found, *ZmGCN5*, which is expressed in all tissues of the plant, is localized in the nucleus, and interacts with the *AtADA2b* homolog ZmADA2b [128]. For maize, we only know that histone acetylation plays a role in the cell cycle. Sodium butyrate treatment causes cell cycle arrest at preprophase in the meristematic zone of *Z. mays* by increasing the total level of acetylated K9 in histone H3 and K5 in histone H4, accompanied by ROS-mediated DNA topoisomerase inhibition [129]. The underlying mechanism could be the same as is in the endosperm, where ZmRpd3 proteins are involved in cell cycle progression through interacting with the maize retinoblastoma-related protein, ZmRBR1, a regulator of the G1/S transition, and with the maize retinoblastoma-associated protein, ZmRbAp1, a histone-binding protein involved in nucleosome assembly [130,131,132]; however, this hypothesis needs to be further tested.

In poplar (*P. trichocarpa*), a genome-wide analysis of the HAT and HDA genes found 12 HAT and 16 HDA genes [133]. Promoter element analysis as well as expression analysis showed that both HDA and HAT genes are responsive to environmental stimuli, such as drought and multiple hormones, such as ABA, MeJA, and SA [133,134]. Interestingly, the expression of both HAT and HDA genes has been found to be extremely low in the root. This could reflect a lack of significant regulatory roles for HATs and HDACs in the root or could potentially reflect expression of the genes in specific cell types, regulating cell-type-specific functions. In poplar, there are two *AtGCN5* homologous genes (*PtrGCN5-1* and *PtrGCN5-2*) and one *AtADA2b* gene (*PtrADA2b*) [134]. *PtrGCN5-1* is drought-inducible and abundantly expressed in xylem tissues but *PtrGCN5-2* is not inducible and expressed at much lower levels [134], suggesting that the two genes could be specialized in different functions. TSA treatment in *P. trichocarpa* mirrors the inhibitory effects observed in *A. thaliana* and causes primary root growth inhibition and delayed adventitious root generation. This further supports the notion that HDAs might have divergent functions depending on the plant species. In addition to the delayed root generation, the generated roots appear much thicker than the control roots due to increased cortex cells. In contrast, cell size remains unaffected. Moreover, TSA-treated plants exhibited a differential gene expression profile, where most genes were downregulated. This finding was the first to highlight the role of HDAs in root organogenesis, growth, and development in poplar [135]. This was further supported when HISTONE DEACETYLASE 902 (PtHDT902) was characterized as an essential regulator in poplar’s adventitious root formation. Overexpression of *PtrHDT902* inhibits adventitious root formation in poplar and enhances primary root growth in *A. thaliana*. *PtHDT902* overexpression in both *A. thaliana* and poplar increases the expression levels of gibberellin (GA) biosynthesis genes, which explains why the *PtrHDT902* overexpression phenotypes are consistent with the GA overproduction phenotypes [136]. Recently, it was also shown that PtrWOX4a and PtrVCS2 form a regulatory nexus with a histone modification system to regulate normal vascular cambium development for wood formation in *P. trichocarpa* [137].

Ultimately, even though the past few years of research regarding the effect of histone post-translational modifications on root development of crop plant species has increased, compared to *A. thaliana*, only a few histone acetylation- and deacetylation-related genes are well-characterized and affect root development amongst these species (Table 2).

## 9. Summary, Challenges, and Future Perspectives

Histone acetylation and deacetylation are common, conserved mechanisms that regulate chromatin structure, control gene expression, and affect various aspects of plant development under normal and stress conditions. The involvement of histone acetylation in root development includes the regulation of root zonation through fine-tuning the distinct cellular activities that characterize each zone, such as stem cell maintenance, cell cycle progression, cell division, expansion, and differentiation. There is diverse evidence supporting that histone acetylation regulates the expression of specific genes, which are key modulators of several root development processes; however, future research should clarify whether the effects of histone acetylation on root development are direct or mediated through indirect pathways. Researchers could also focus on other members of the HAT and HAD families that are underrepresented in the developmental studies. It is also evident that histone acetylation and deacetylation may have different effects on root development across several plant species; however, in order to verify this, investigation of histone acetylation in other model plant species and economically important crops, such as legumes or Brassica, should be carried out and the findings could also bridge the gap between pure and applied research.

Research on histone acetylation and deacetylation in plants admittedly faces some challenges, particularly due to the pleiotropic effects of mutations in HAT and HDA genes, which impact several aspects of plant development. To better understand the role of histone acetylation in root-specific developmental processes, creating tissue- or organ-specific mutants could provide more targeted insights. Also, relatively few histone modulators have been well-studied, which raises the need for more HAT and HDA family members to be investigated in detail. In particular, since histone acetylation is a dynamic process, the equilibrium between histone acetylation and deacetylation should be further assessed to determine the relationship and orchestration between them during the regulation of gene transcription. In addition, in vitro systems have been dominantly used for root-related research. While they have been instrumental in advancing our knowledge regarding root development and responses, the non-natural conditions—namely the light exposure of roots—can lead to biases or misinterpretations of the experimental results. Light can alter root development, stress responses, and interactions with microorganisms, making it crucial to refine experimental setups. For this reason, improved systems that achieve simulating natural root environments without illumination have been proposed, such as the Rhizotrons [138], the D-Root [139], the GLO-Roots [140], and the Rhizobox systems [141], and need to be carefully considered as a possible step forward.

In addition to the technical challenges, several areas of root development regulation by histone acetylation remain underexplored, such as the involvement of H4 acetylation, non-histone protein acetylation, and histone variants. Vascular tissue development, secondary root growth, and lateral root formation also need deeper investigation as they are important for structural support, water and nutrient transportation, and enhancement of root surface area. Moreover, the crosstalk between histone modifications needs to be examined more carefully by the incorporation of multi-omics approaches as the specific pattern of post-translational histone modifications can influence gene expression. Complex relationships between acetylation, methylation, phosphorylation, and ubiquitination during root development might exist, and possible regulatory feedback loops need to be identified.

Forward and reverse genetic approaches can be complemented by new genetic tools and techniques in order to tackle the aforementioned challenges. Integrating methods such as spatial transcriptomics, bulk RNA-seq, ChIP-seq, proteomics, and phospho-proteomics will allow researchers to link transcriptional activity to protein translation, providing a more comprehensive understanding of how histone modifications shape plant phenotypes. Additionally, the use of innovative techniques, such as single-cell or single-nucleus RNA-seq can illuminate the small, yet biologically significant, transcriptional changes within individual root cell types; however, the interpretation of the data needs to be performed with caution because the cell dissociation techniques that are used prior to sequencing induce stress responses and can alter gene expression. Moreover, the CRISPR/Cas9 genome editing tool can be used to create mutations in genes of interest, both in *A. thaliana* and in other plant model organisms or crops, to further study the effect of histone acetylation in root development. Other than gene knockouts or knockdowns, CRISPR/Cas9 technology can be used to create scar-free, stable, and heritable gene knock-ins for plants [142], which unlocks a lot of potential for research on functional genomics and evolutionary conservation or divergence of genes between *A. thaliana* and other plant species. Concomitantly, the knowledge generated in model plants like *A. thaliana* will be translated to economically important crops, to ensure that these fundamental insights can be applied to improve agricultural practices and crop resilience.

## Figures and Tables

**Table 1:** Number of HAT and HDA genes in *Oryza sativa*, *Zea mays,* and *Populus trichocarpa*.

Molecular Function	Family Name	*Arabidopsis thaliana*	*Oryza sativa*	*Zea mays*	*Populus trichocarpa*
Histone acetyltransferases	GNAT	4	3	2	5
	MYST	2	1		2
	CBP	5	3		3
	TAF_II_250	2	1		2
Histone deacetylases	RPD3/HDA1	12	14	12	11
	SIR2	2	2	2	2
	HD2	4	2	4	3

**Table 2:** Histone acetylation- and deacetylation-related genes affect root development in different plant species.

Organism	Gene Symbol	Locus ID	Molecular Function	Role in Root Development
*Arabidopsis thaliana*	*AtADA2b*	AT4G16420	Transcriptional co-activator	Stem cell niche maintenance [59,68] Meristem size [56,68] Root zonation [59,61,68,117]
	*At* *GCN5*	AT3G54610	Histone acetyltransferase	Stem cell niche maintenance [59,68] Meristem size [59,68] Cell patterning in root epidermis [102] Root zonation [59,61,68,117]
	*AtHAF2*	AT3G19040		Cell patterning in root epidermis [102]
	*AtHDA6*	AT5G63110	Histone deacetylase	Cell patterning in root epidermis [101]
	*AtHDA18*	AT5G61070		Cell patterning in root epidermis [99,100]
	*AtHDA19*	AT4G38130		Root cell elongation [95] Cell patterning in root epidermis [102,103] Differentiation of the columella cells [97]
	*AtHD2A* or *AtHDT1*	AT3G44750		Switch from cell division to expansion [93]
	*AtHD2B* or *AtHDT2*	AT5G22650		
	*AtHD2D* or *HDT4*	AT2G27840		Lateral root development [111,112,113]
	*AtLDL1* or *AtSWP1*	AT1G62830	Histone demethylase	Fine-tuning of root elongation [94] Lateral root development [108,109] Component of a putative HDA co-repressor complex [94]
*Oryza sativa*	*OsADA2*	LOC_Os03g53960	Transcriptional co-activator	Cell division and growth [123] Energy metabolism [123] Cell wall biosynthesis [123] Hormone response [123]
	*OsGCN5*	LOC_Os10g28040	Histone acetyltransferase	Cell division and growth [123] Energy metabolism [123] Cell wall biosynthesis [123] Hormone response [123]
	*OsHDAC1*	LOC_Os06g38470	Histone deacetylase	Root growth [120,121] Lateral root formation through HDAC1/GSK2/BZR1 module [122]
*Populus trichocarpa*	*PtrHDT902*	Potri.009G170700	Histone deacetylase	Adventitious root formation [136]
	*PtrGCN5-1*	Potri.002G045900	Histone acetyltransferase	Vascular cambium development [137]
	*PrtADA2b-3*	Potri.004G135400	Transcriptional adaptor	

## Data Availability

The corresponding author K.V. is responsible for distribution of the materials generated in this study in accordance with the policy described in MDPI Research Data Policies at https://www.mdpi.com/ethics (accessed on 29 September 2024).
